# Tetrandrine Suppresses Human Brain Glioblastoma GBM 8401/*luc2* Cell-Xenografted Subcutaneous Tumors in Nude Mice In Vivo

**DOI:** 10.3390/molecules26237105

**Published:** 2021-11-24

**Authors:** Ching-Lung Liao, Yi-Shih Ma, Te-Chun Hsia, Yu-Cheng Chou, Jin-Cherng Lien, Shu-Fen Peng, Chao-Lin Kuo, Fei-Ting Hsu

**Affiliations:** 1 School of Chinese Medicine, College of Chinese Medicine, China Medical University, Taichung 40604, Taiwan; qbking@ms29.hinet.net; 2 School of Chinese Medicine for Post-Baccalaureate, I-Shou University, Kaohsiung 840, Taiwan; m2367591@ms25.hinet.net; 3 Department of Chinese Medicine, E-Da Hospital, Kaohsiung 824, Taiwan; 4 Department of Respiratory Therapy, China Medical University, Taichung 404, Taiwan; derrick.hsia@msa.hinet.net; 5 Department of Internal Medicine, China Medical University Hospital, Taichung 404, Taiwan; 6 Department of Neurosurgery, Neurological Institute, Taichung Veterans General Hospital, Taichung 407, Taiwan; chouycns@yahoo.com.tw; 7 Department of Neurological Surgery, Tri-Service General Hospital, National Defense Medical Center, Taipei 114, Taiwan; 8 School of Pharmacy, China Medical University, Taichung 404, Taiwan; jclien@mail.cmu.edu.tw; 9 Department of Medical Research, China Medical University Hospital, Taichung 404, Taiwan; t20811@mail.cmuh.org.tw; 10 Department of Biological Science and Technology, China Medical University, Taichung 404, Taiwan; 11 Department of Chinese Pharmaceutical Sciences and Chinese Medicine Resources, China Medical University, Taichung 404, Taiwan

**Keywords:** Tetrandrine (TET), human brain glioblastoma GBM 8401/*luc2* cells, xenograft tumor, nude mice, in vivo

## Abstract

Tetrandrine (TET), a bisbenzylisoquinoline (BBI) alkaloid, is isolated from the plant *Stephania tetrandra* S. Moore and has a wide range of biological activity, including anticancer properties in vitro and in vivo. At first, we established a luciferase-expressing stable clone that was named GBM 8401/*luc2* cells. Herein, the primary results indicated that TET reduced the total cell viability and induced cell apoptosis in GBM 8401/*luc2* human glioblastoma cells. However, there is no available information showing that TET suppresses glioblastoma cells in vivo. Thus, we investigated the effects and mechanisms of TET on a GBM 8401*/luc2* cell-generated tumor in vivo. After the tumor volume reached 100–120 mm^3^ in subcutaneously xenografted nude mice, all of the mice were randomly divided into three groups: Group I was treated with phosphate-buffered solution (PBS) containing 0.1% dimethyl sulfoxide, Group II with 25 mg/kg of TET, and Group III with 50 mg/kg of TET. All mice were given the oral treatment of PBS or TET by gavage for 21 days, and the body weight and tumor volumes were recorded every 5 days. After treatment, individual tumors, kidneys, livers, and spleens were isolated from each group. The results showed that TET did not affect the body weights, but it significantly decreased the tumor volumes. The TET treatment at 50 mg/kg had a two-fold decrease in tumor volumes than that at 25 mg/kg when compared to the control. TET decreased the total photon flux, and treatment with TET at 50 mg/kg had a lower total photon flux than that at 25 mg/kg, as measured by a Xenogen IVIS imaging system. Moreover, the higher TET treatment had lower tumor volumes and weights than those of the lower dose. The apoptosis-associated protein expression in the tumor section was examined by immunohistochemical analysis, and the results showed that TET treatment reduced the levels of c-FLIP, MCL-1, and XIAP but increased the signals of cleaved-caspase-3, -8, and -9. Furthermore, the hematoxylin and eosin (H & E) staining of kidney, liver, and spleen tissues showed no significant difference between the TET-treated and control groups. Overall, these observations demonstrated that TET suppressed subcutaneous tumor growth in a nude-mice model via the induction of cell apoptosis.

## 1. Introduction

Gliomas, which are the most frequently diagnosed intracranial malignancies that originate in the brain parenchyma, have high morbidity and mortality worldwide [1]. Human gliomas include six subtypes: adult-type diffuse gliomas, pediatric-type diffuse low-grade gliomas, pediatric-type diffuse high-grade gliomas, circumscribed astrocytic gliomas, glioneuronal and neuronal tumors, and ependymomas. Therefore, based on the World Health Organization’s Classification of Tumors of the Central Nervous System, which was proposed in 2021, a glioblastoma (GBM) belongs to the subtype of adult-type diffuse gliomas [2] and is the most biologically aggressive, frequent, and lethal subtype of malignant gliomas [3]. The mean survival time of GBM patients is only approximately 15 months, even after systematic treatments [4,5]. Currently, the clinical therapy for GBM patients is surgical resection followed by adjuvant radiation therapy and chemotherapy with oral temozolomide (TMZ). However, radiation and TMZ (the standard chemotherapeutic agent) chemotherapy for GBM patients show a 9.8% chance of a five-year survival [6], and it only increases the likelihood of a two-year survival from 10% to 26% [7]. Unfortunately, >90% of GBM tumors recur at the original site [8]. TMZ has been shown to induce significant side effects (clinical toxicity) [9]. Thus, the clinical need to find new drugs for GBM therapeutics is urgent.

Tetrandrine (TET), a bisbenzylisoquinoline alkaloid, is isolated from the dried roots of the traditional Chinese medicinal herb *Stephania tetrandra* S. Moore [10] and other related species of *Menispermaceae* [11]. It possesses anti-tumor activity in vitro and in vivo against many types of human cancers such as breast, liver, pancreatic, leukemia, lung, prostate, gastric, and colorectal cancer [12,13,14,15,16,17]. TET was confirmed to act as a calcium-channel blocker and against silicosis, hypertension, inflammation, and cancer without any toxicity [11]. TET caused cell cycle arrest in the G1 phase in colon cancer cells [18], induced mitochondria-mediated apoptosis in human gastric cancer BGC-823 cells [19], and triggered autophagy in human breast cancer MDA-MB-231 cells through the suppression of PI3K/AKT/mTOR signaling [20]. TET also suppressed the migration and invasion of human colon cancer SW620 cells via down-regulation of nuclear factor-kappaB and the matrix metalloproteinase signaling pathways [21]. In addition, TET was shown to suppress tumor growth and the angiogenesis of gliomas in rats [22,23]. Furthermore, it exhibited a reversal of drug resistance by modulating P-glycoprotein levels [24]. TET has been found to potently increase the transport across the blood-brain barrier (BBB) by inhibiting the P-glycoproteins that are overexpressed on the BBB [25,26,27]. 

In our earlier studies, we showed that TET induced cell apoptosis in human nasopharyngeal carcinoma NPC-TW 076 cells via endoplasmic reticulum stress and caspase-dependent signaling pathways [28,29], and in NPC-TW 039 cells through endoplasmic reticulum stress and Ca^2+^/calpain pathways [30]. It also induced cell apoptosis and autophagy in human oral cancer HSC-3 cells [31]. TET caused apoptotic cell death in human neuroblastoma by regulating the Hippo/YAP signaling pathway [32]. Recently, we also found that TET inhibited cell migration and invasion of human brain glioblastoma GBM 8401 cancer cells [33]. However, there is no report showing that TET affects GBM 8401 cancer cells in vivo; thus, the purpose of the present study is to focus and investigate the effects of TET on the GBM 8401/*luc2*-cell-xenografted mice model in vivo. The results indicated TET induced apoptotic cell death in the luciferase-expressing stable clone that was named GBM 8401/*luc2* cells, and significantly suppressed and reduced tumor growth in vivo. 

## 2. Results

### 2.1. The Effects of TET on Cell Viability and Apoptosis in GBM 8401/luc2 Cells

**Figure 1:**
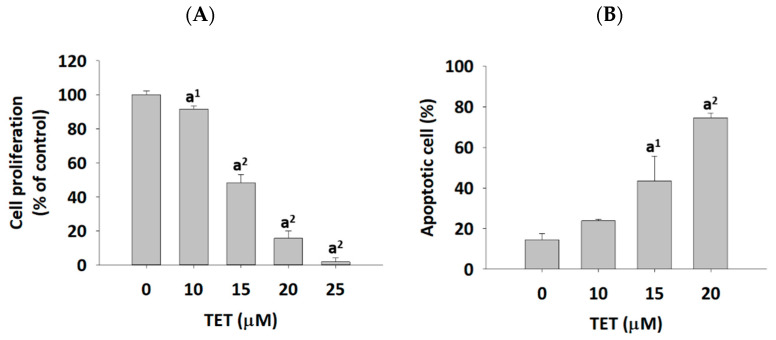
The effect of TET on cell viability and apoptosis in GBM 8401/*luc2* cells. Cells (1 × 10^5^ cells/well) were incubated with TET (0, 10, 15, 20, and 25 μM) for 48 h, and cells were harvested and measured for cell viability (**A**) and apoptosis (**B**) using a flow cytometer as described in Materials and Methods. Data are expressed as mean ± standard error. Comparisons between control and TET-treated groups were performed using one-way ANOVA. (a^1^ *p* < 0.05 and a^2^ *p* < 0.01 vs. control).

GBM 8401/*luc2* cells were treated with TET, and the cells were harvested for measuring the cell viability and apoptosis using a flow cytometer, and the results are shown in Figure 1. TET at 10–25 μM significantly decreased 8.35–98.16% of the total viable cell number (cell viability) (Figure 1A); however, TET at 10–20 μM induced apoptotic cell death in 23.81–74.59% of cells (Figure 1B). These effects are dose-dependent. 

### 2.2. TET Affected Body Weights and Glioblastoma Tumor Growth

**Figure 2:**
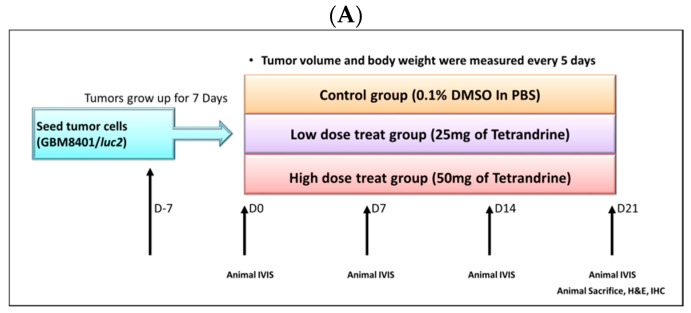

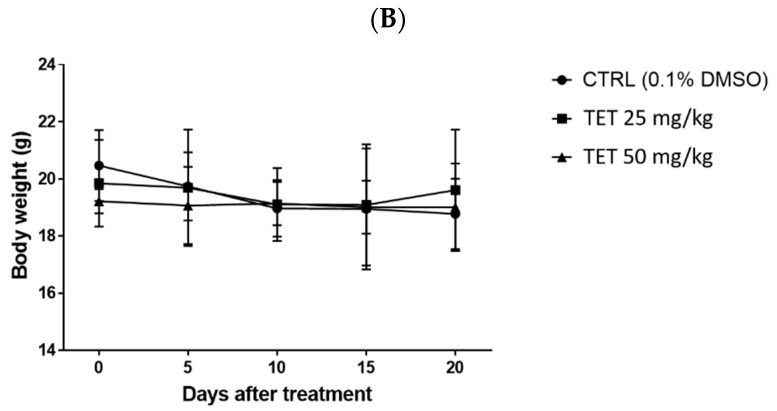
The flow chart and body weight investigation of TET-treated animal experiment in xenografted GBM 8401/*luc2*-cell-bearing animals. An animal experiment flow chart was displayed (**A**), and the body weights of mice from each group were measured every five days (**B**).

In order to investigate the anti-tumor activity of TET in vivo, the glioblastoma (GBM 8401/*luc2* cells) subcutaneous xenograft models were established. The flow chart of the whole animal experiment is displayed in Figure 2A. TET was used to treat glioblastoma-bearing mice for 21 days, the body weight of each mouse was recorded, and the mouse tumor size was measured using a caliper every five days. Average body weights from each group are presented in Figure 2B. The results indicate that TET did not significantly affect the body weights of these groups of mice.

**Figure 3:**
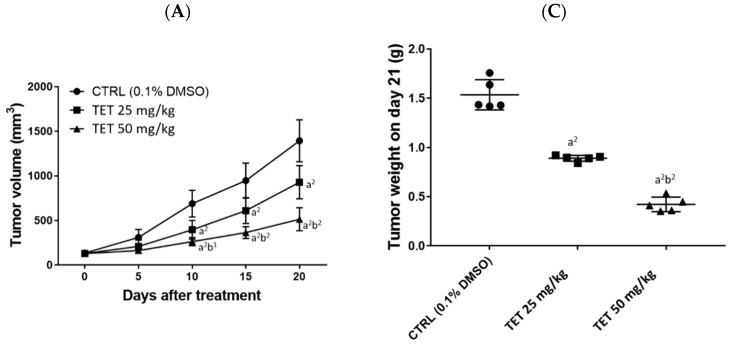

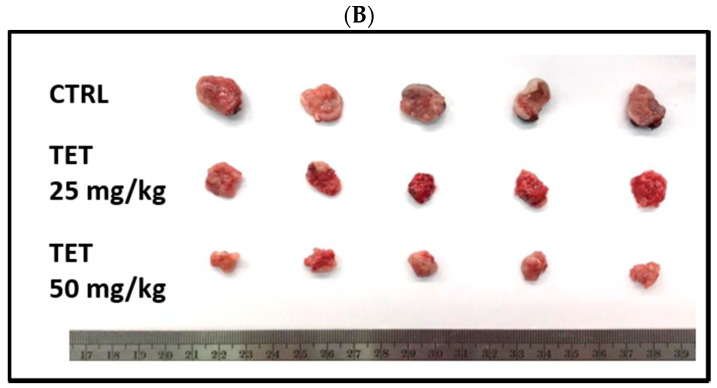
TET suppressed the tumor growth in GBM 8401/*luc2*-cell-xenografted animals. Tumor volume was measured by caliper every five days and quantified (**A**). Tumors from each group of mice were isolated and represented (**B**) and tumor weight was also measured and quantified (**C**) on day 21. (a^2^ < 0.01 vs. control; b^1^ *p* < 0.05 and b^2^ < 0.01 vs. TET 25 mg/kg).

The average tumor volume from each group was measured every five days, and the results are shown in Figure 3A. At the end of treatment, all of the mice were sacrificed and the tumors were individually isolated. The representatives of tumors and tumor weights from each group are shown in Figure 3B,C, respectively. Based on these observations, TET effectively inhibited the growth of mouse tumors (tumor volume), and a significant difference was found after day 10 for both doses (25 and 50 mg/kg) of TET treatment (Figure 3A). The statistical analysis results of the tumor volumes between groups are displayed in Table 1 and Table 2. Figure 3B,C show that after the tumors were weighed on day 21, both doses of TET (25 and 50 mg/kg) had significantly reduced their weight, and the higher dose (50 mg/kg) of TET had more significant inhibition than that of the low dose (25 mg/kg) in the examined animals.

### 2.3. TET Significantly Reduced the Signal Intensity of Luc2 from the Subcutaneous Glioblastoma-Bearing Mice

**Figure 4:**
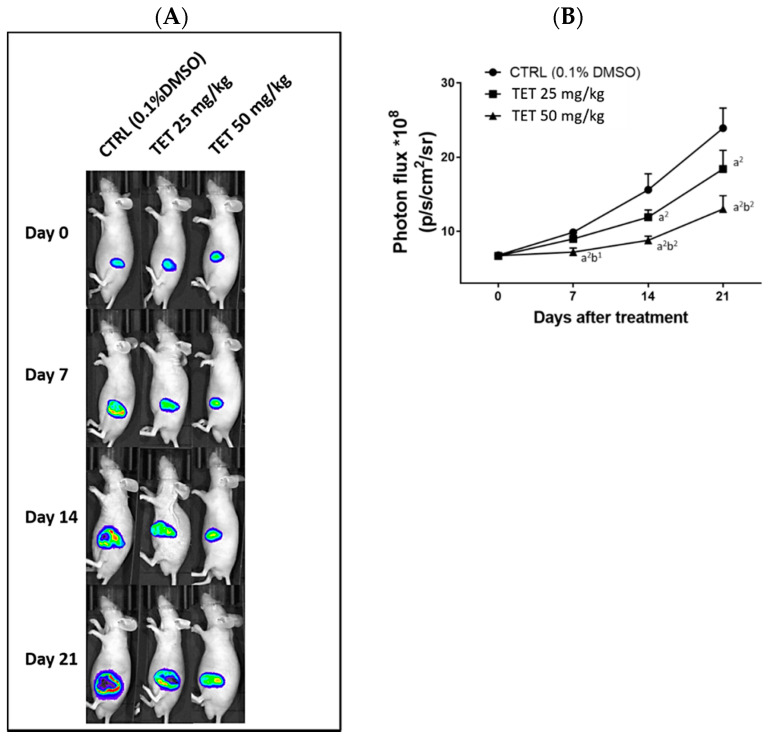
TET-suppressed *Luc2* signals from living tumor cells. Animals were injected with GBM 8401/*luc2* cells and *Luc2* signals of the tumors from each group were further measured. The representative BLI results from each group at different time points (**A**). Quantification results of *Luc2* signal intensity of tumors (**B**). (a^2^ < 0.01 vs. control; b^1^ *p* < 0.05 and b^2^ < 0.01 vs. TET 25 mg/kg).

After the GBM 8401/*luc2* cells were injected into each mouse, the *Luc2* signal of the tumor, which represented tumor growth, was collected by BLI every week. All of the represented bioluminescent images of mice from each group on days 0, 7, 14, and 21 are shown in Figure 4. The *Luc2* signal intensity of the control group at day 21 of treatment was enhanced by about 150 times than that at day 0. Both dosages (25 and 50 mg/kg) of TET effectively decreased the *Luc2* intensity growth in glioblastoma tumors when compared to the control. Moreover, the high dosage (50 mg/kg) of TET showed superior tumor (*Luc2* signal) inhibition capacity than that of the low dosage of TET (25 mg/kg). The statistical analysis results of the tumor photon flux between groups are displayed in Table 3. Furthermore, these results were consistent with the results of the tumor volume and weight.

### 2.4. TET Affected Anti-Apoptosis and Pro-Apoptosis Factors in Glioblastoma-Bearing Mice

**Figure 5:**
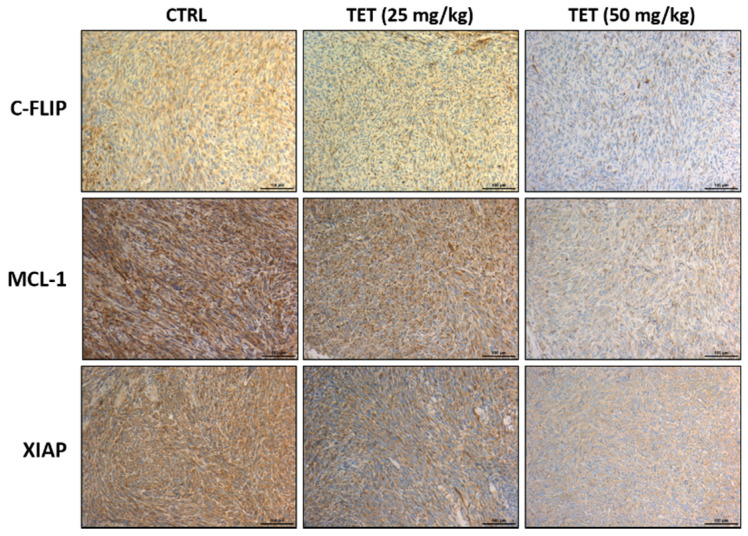
TET inhibited the levels of anti-apoptosis-related proteins. Tumors were isolated from each group and further examined by IHC staining. The IHC staining images of c-FLIP, MCL-1, and XIAP were observed under a microscope with 100 times magnification. All processes were described in Materials and Methods. Scale bar = 100 μm.

After treatment at day 21, the mice from each group were sacrificed, and individual tumors were isolated for further protein expression validation. IHC staining was used to investigate the alteration of apoptosis-related markers after TET treatment. The expression of c-FLIP, MCL-1, and XIAP, which have been well documented to be anti-apoptosis markers and act as suppressed tumor apoptosis, are shown in Figure 5. After TET treatment, the protein levels of c-FLIP, MCL-1, and XIAP were decreased compared to the control (Figure 5). TET has been shown to induce apoptosis in cancer cells.

**Figure 6:**
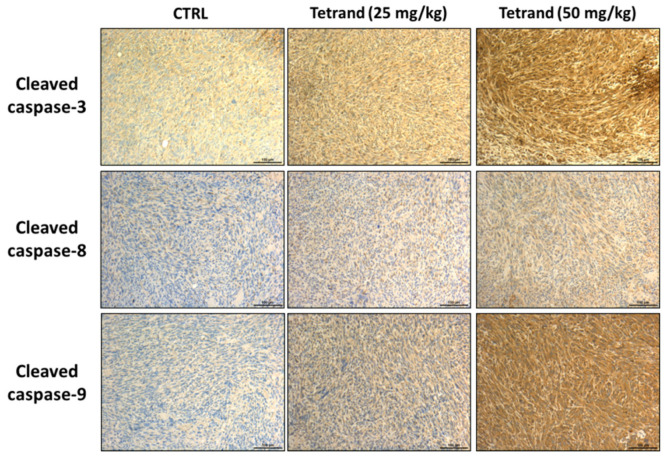
TET increased the expressions of pro-apoptosis-related proteins. Tumors were isolated from each group and further examined by IHC staining. The IHC staining images of cleaved caspase-3, -8, and -9 were observed under a microscope with 100 times magnification. All processes were described in Materials and Methods. Scale bar = 100 μm.

Thus, we further examined whether TET affected the apoptosis-associated protein expressions such as caspase-3, -8, and -9, and the results are shown in Figure 6. TET increased the levels of cleaved caspase-3, -8, and -9 compared to the control group (Figure 6). Based on these results, we suggest that TET may suppress tumor growth via the regulation of the apoptosis signaling of glioblastoma in vivo.

### 2.5. TET Treatment Affected Acute or Decreased Toxicity of Glioblastoma-Bearing Mice

**Figure 7:**
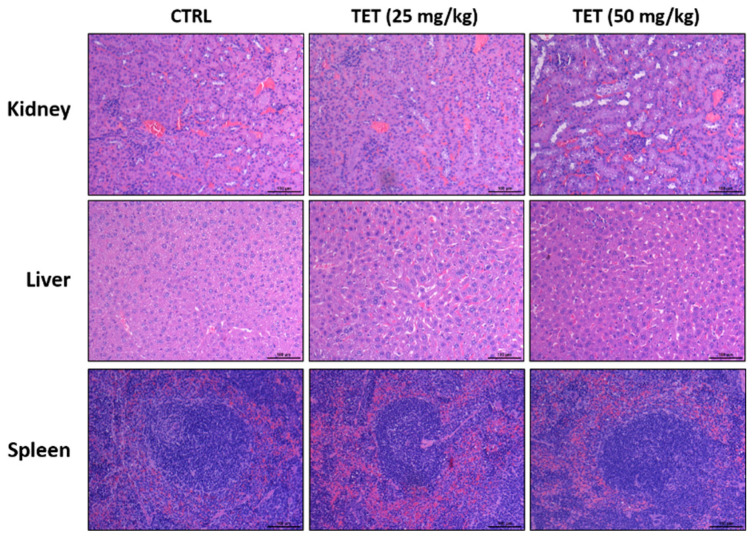
The examinations of TET-induced acute toxicity from xenografted GBM 8401/*luc2*-cell-bearing mice. After treatment, tissues such as kidney, liver, and spleen were isolated from each mouse and further examined the pathology photograph by H & E staining under the microscope with 100 times magnification as described in Materials and methods. Scale bar = 100 μm.

A macroscopic examination of vital organs, including liver, kidney, and spleen, was carried out soon after the animals were sacrificed. Histopathological observation consisted of monitoring tissue integrity and searching for injuries such as degeneration, necrosis, and apoptosis, which indicate signs of toxicity. For further investigation into whether or not the dosage of TET (25 and 50 mg/kg) causes the toxicity of mice, H & E staining on mice kidney, liver, and spleen was performed and the results are shown in Figure 7. The acute toxicity can damage some organs or tissues, resulting in pathological changes of the tissues; however, in the present studies, there were no significant pathological differences in the kidney, liver, and spleen tissues among the three administration groups. Besides, the increase or decrease in the body weight of animals is an indicator of an adverse effect of drugs. The body weight results from Figure 2B also show no significant difference between the TET-treated and control groups. Based on these observations, there are no signs of acute or decreased toxicity in these treatment animals. 

## 3. Discussion

Glioma has been recognized as a deadly malignancy and accounts for about 80% of primary brain cancers [34]. Glioblastoma (GBM) is a subtype of glioma, and the current treatment for patients with GBM is still insufficient. Numerous studies have attempted to improve the outcome of GBM, and some of the reports focused on natural products and exploring the molecular mechanisms underlying the tumor progression of GBM. TET presents anti-tumor activity in numerous types of cancer in vitro and in vivo [17,18]. It has been shown to induce cell apoptosis in many human cancer cells [28,29,30,31], including human neuroblastoma [32]. Our earlier studies found that TET inhibits cell migration and invasion in human glioblastoma GBM 8401/*luc2* cells [33]. However, there is no available information showing that TET affects GBM 8401/*luc2* cells in vivo. In the present study, we showed that TET significantly reduced the total cell viability (Figure 1A) and induced cell apoptosis (Figure 1B) in GBM 8401/*luc2* cells; thus, we evaluated the efficacy of TET against GBM 8401/*luc2* cells in vivo through the use of athymic CAnN.Cg-*Foxn1^nu^*/CrlNarl nude mice that were inoculated with GBM 8401/*luc2* cells. We further examined the inhibitory effects of TET on the growth of GBM 8401/*luc2* cells bearing xenografted tumors. The whole flow chart of the experiment outline is shown in Figure 2A. 

After the animals were prepared for the experiment, all of the mice from each group were treated separately with TET at 0, 25, and 50 mg/kg, respectively. The individual body weight was recorded, the tumor volume (size) was measured using a caliper every five days, and the *Luc2* signal intensity of the tumor in each mouse was acquired from BLI every week on glioblastoma-bearing mice, up to 21 days. 

At the end of treatment, all of the mice were sacrificed and their tumors were isolated. Then, the isolated tumors were photographed and weighed for each mouse of each group. Furthermore, tissues such as the kidney, liver, and spleen were also isolated for the investigation of toxicity experiments. The results indicated that TET at both doses (25 and 50 mg/kg) did not significantly affect body weights (Figure 2B), and both doses significantly reduced tumor volume compared to the control; the representative tumor is pictured in Figure 3A. Furthermore, the higher dose of TET showed a higher inhibition of tumor volume (Figure 3A) and weights (Figure 3C) than that of the lower dose. 

The Xenogen IVIS imaging system is suitable for measuring the effects of test chemicals on tumor growth in xenografted animal models [35,36]. Mice were inoculated subcutaneously with GBM 8401/*luc2* cells and then randomly separated into control, 25, and 50 mg/kg of TET-treated groups. The individual tumors of the mice from each group were monitored in order to detect the photons that they emitted. The *Luc2* signal intensity from each mouse of each group was detected and photographed at day 0, 7, 14, and 21 of treatments using the Xenogen IVIS imaging system. The represented bioluminescent images and the total *Luc2* signal intensity of mice are displayed and calculated in Figure 4. 

Based on the results from Figure 4, which indicate that the *Luc2* signal intensity from the control group was increased after treatment, and that at day 21 of treatment it was enhanced almost 150-fold compared to that at day 0 (Figure 4B). Both doses of TET significantly reduced the *Luc2* intensity growth compared to the control on days 14 and 21 in glioblastoma tumors (Figure 4B). Furthermore, a higher dose (50 mg/kg) of TET treatment has a higher inhibition of *Luc2* signal intensity than that of the lower dose (25 mg/kg). These results are also in agreement and consistent with the tumor volume results (Figure 3); thus, these data indicate that TET at both dose treatments significantly inhibited tumor growth, including tumor volumes and weights in vivo (Figure 3). The results also show that the higher dose of TET had a higher inhibition of tumor growth in vivo. Therefore, we may suggest that TET is a potential candidate for the development of anti-human-brain-tumor drugs in the future.

For further investigation, the effects of TET on the inhibition of GBM 8401/*luc2*-cell-xenografted tumors in nude mice were mediated by the pro-apoptotic and/or anti-apoptotic signaling pathways. Thus, all tumors were collected, stained, and examined by immunohistochemical analysis. Immunohistochemistry has become an essential and critical tool for classifying tumors and has the potential to improve the histopathological diagnosis of neoplasms. Our experimental design is consistent with the effects of frontline chemotherapeutic agents, which have been shown to induce cancer cell apoptosis [37]. Thus, the anti-apoptotic proteins such as c-FLIP, MCL1, and XIAP and pro-apoptotic proteins such as cleaved caspase-3, -8, and -9 were selected as target proteins for the examination of the protein expressions by immunohistochemical analysis, and the results are shown in Figure 4 and Figure 5. As cells begin to undergo apoptosis, the caspases such as caspase-3, -8, and -9 will be activated, and their pro-caspase levels will be decreased via the formation of cleaved-caspases [38]. The expression of cleaved caspase-3, -8, and -9 were monitored in tumor tissues, and the results indicate that TET treatment increased the levels of cleaved caspase-3, -8, and -9 in the tumor section. Caspases play a crucial and central role in the modulation of apoptosis. Caspase-8 is an initiator for the Fas-induced death pathway; caspase-9 involves mitochondria-mediated cell death [39]; caspase-3 is the effector caspase and is vital for both cell-death pathways [40]. Moreover, the inhibition of cancer cell proliferation followed by the induction of cancer cell death via apoptosis has been recognized to be one of the best strategies for cancer treatment [41,42]. 

Activated caspases and the proteasome could cause reduced levels of c-FLIP (a master of apoptotic regulators [43], MCL-1 (anti-apoptotic members of BCL-2) [44], and XIAP (anti-apoptotic protein) [45,46,47,48]. Herein, the results from Figure 5 indicate that TET significantly inhibited the expressions of c-FLIP, MCL-1, and XIAP in the tumor tissues of the GBM 8401-cell-xenografted mice. Moreover, both the synthesis of MCL-1 and c-FLIP (both are short-lived proteins) have been regulated by mTOR signaling [49]. XIAP is an inhibitor of cell death that is involved in the inhibition of specific caspases [50], and it also mediates chemotherapy resistance and apoptosis resistance [51]. 

We found that oral administration of TET (25 and 50 mg/kg) reduced tumor size (volume) and weights in the GBM 8401/*luc2*-cell-xenografted animals (Figure 3), but it did not significantly affect their body weights (Figure 2B). With regard to further investigation and examination of the cytotoxic effects of TET, we observed the H & E staining of kidney, liver, and spleen tissues, and the results indicate that TET did not induce cytotoxic effects in these tissues. Furthermore, TET suppressed tumor volumes and sizes by the induction of cell apoptosis based on the markedly increased active forms of caspase-3, -8, and -9 in the TET-treated groups. Therefore, our findings may provide additional TET targets. On the other hand, our findings regarding the effects of TET on the GBM subcutaneous animal model and its anti-glioma capacity need to be confirmed in the orthotopic model in the future.

## 4. Materials and Methods 

### 4.1. Chemicals and Reagents

Tetrandrine (TET) and dimethyl sulfoxide (DMSO) were bought from Sigma Chemical Co. (St. Louis, MO, USA), and TET was dissolved in dimethyl sulfoxide (DMSO) as 150 mg/mL stock. Hygromycin B was obtained from Santa Cruz Biotechnology (Dallas, TX, USA). Roswell Park Memorial Institute (RPMI) 1640 Medium and penicillin-streptomycin were purchased from Life Technologies (Carlsbad, CA, USA). Heat-inactivated fetal bovine serum (FBS) was obtained from Hyclone Laboratories (Logan, UT, USA), D-luciferin and pGL4.50 luciferase reporter (pGL4.50 [*luc2*/CMV]) vector from Promega (Madison, WI, USA), and JetPEI™ transfection reagent from Polyplus Transfection (Illkirch, Bas-Rhin, France). Primary monoclonal antibody anti-c-FLIP (1:300 dilution), anti-MCL-1 (1:300 dilution), anti-cleaved caspase-3 (1:300 dilution), anti-cleaved caspase-8 (1:300 dilution), and anti-cleaved caspase-9 (1:300 dilution) were obtained from Cell Signaling Technology (Danvers, MA, USA), and anti-XIAP from Elabscience Biotechnology Inc. (1:300 dilution; Houston, TX, USA).

### 4.2. Cell Culture of Human Glioblastoma GBM8401 Cells

A human glioblastoma cell line (GBM 8401 with p53 mutation and pTEN wild type) [52,53] was obtained from the Food Industry Research and Development Institute (Hsinchu, Taiwan). GBM 8401 cells were plated in 6-cm culture dishes containing RPMI-1640 medium with 10% heat-inactivated fetal bovine serum (FBS), 2 mM L-glutamine, and antibiotics (100 U/mL penicillin and 100 μg/mL streptomycin) in an incubator at 37 °C with a humidified atmosphere of 5% CO_2_ [54]. 

### 4.3. Cell Transfection and Stable Clone Selection in GBM 8401 Cells

About eighty percent of the confluence of GBM 8401 cells on the 6-cm dishes was reached before the transfection procedure. The detailed transfection process and selection methods of stable clones were performed as previously described [55]. At the end of transfection, the *Luc2* expression in GBM 8401 cells was screened with hygromycin B (200 μg/mL), and GBM 8401 cells with *Luc2* signals were selected by IVIS 200 Imaging System (Xenogen, Alameda, CA, USA) and identified as GBM 8401/*luc2* cells.

### 4.4. Cell Viability Assay

GBM 8401/*luc2* cells (1 × 10^5^ cells/well) were seeded into 24-well culture plates for 24 h and incubated with TET at the final concentrations (0, 10, 15, 20, and 25 μM) for 48 h. After treatment, cells from each well were collected, resuspended in PBS containing 5 μg/mL of PI solution, and then analyzed by flow cytometric assay for measurement of the total viable cell number (cell viability) as described in previous studies [54].

### 4.5. Cell Apoptosis Assay

GBM 8401/*luc2* cells (1 × 10^5^ cells/well) were maintained in 24-well plates for 24 h and treated with 0, 10, 15, and 20 μM of TET for 48 h. At the end of incubation, cells were collected from each treatment and mixed well with Annexin V-FITC/PI solution for 15 min in the dark as per the manufacturer’s instructions. All samples were analyzed by flow cytometric assays for total cell apoptosis as previously described [54]. 

### 4.6. Xenograft GBM 8401-Cell-Bearing Animal Model

Fifteen athymic CAnN.Cg-*Foxn1^nu^*/CrlNarl (NUDE) male animals, 6–8 weeks old, were purchased from the National Laboratory Animal Center (Taipei, Taiwan). All animals were maintained in standard cages at 25 °C with a filtered laminar airflow room in the China Medical University’s animal center (Taichung, Taiwan). The animal experiments were approved by the Institutional Animal Care and Use Committee at China Medical University (CMU-IACUC 2020–248). After 7 days of acclimation, the glioblastoma animals were established by subcutaneously injected GBM 8401/*luc2* cells (1 × 10^7^ cells/mouse) into the right flank of the animals [55]. 

### 4.7. Treatment and Physical Tumor Growth Validation of the Animal Model

The overall outline of treatment is shown in Figure 2A. After the GBM 8401/*luc2* cells were injected into the animal, the tumor size and body weight of the animals were monitored. When the animals’ tumor size reached an average of 100 mm^3^, and the bodyweight did not change by more than 5%, all of the mice were randomly separated into three groups: control (PBS containing 0.1% DMSO), 25 or 50 mg/kg of TET groups. TET was diluted with 100 μL of PBS containing 0.1% DMSO and orally administered each day by gavage for 21 days. The body weight of each animal was weighed and recorded. The tumor size of each animal from the three groups was measured using digital calipers every five days. Tumor volume was calculated by the equation: V = L × W^2^ × 0.523 (where V is the volume, L is the length, and W is the width) as described previously [55]. Finally, tumors from each mouse were removed, photographed, and weighed after 21 days of treatment.

### 4.8. In Vivo Bioluminescent Imaging (BLI) of Tumors

All of the mice from each group were intraperitoneally injected with 150 mg/kg of D-luciferin (Promega, Madison, WI, USA) and then held for 15 min before anesthetization using 1–3% isoflurane for BLI scanning. IVIS 200 Imaging System was used for image acquisition, and then the *Luc2* signal intensity from each mouse was quantified by Living Image software (Version 2.20, Xenogen, Alameda, CA, USA) as previously described [55].

### 4.9. Pathology of Kidney, Liver, and Spleen

At the end of treatment, kidney, liver, and spleen tissues of individual mice were isolated and individually fixed by 10% neutral buffered formalin and then embedded by paraffin. The isolated tissue sections were stained with hematoxylin and eosin (H & E) that were used as an anatomical pathology diagnosis for comparing untreated and TET-treated mice [56]. 

### 4.10. Immunohistochemistry Staining

The expressions of Cellular FLICE (FADD-like IL-1beta-converting enzyme)-inhibitory protein (c-FLIP), myeloid cell leukemia-1 (MCL-1), X-linked inhibitor of apoptosis protein (XIAP), and cleaved caspase-3, -8, and -9 were performed by tumor immunohistochemistry staining as previously described [56]. In brief, the paraffin-embedded tumor section from each animal was mounted on adhesive microscope slides. Tissue sections were deparaffinized, rehydrated in alcohol, and processed using the avidin-biotin immuno-peroxidase method. Then, tissue sections were individually incubated with primary monoclonal anti-c-FLIP, -MCL-1, -cleaved caspase-3, -8, and -9 (Cell Signaling, Beverly, MA, USA) and XIAP (Elabscience Biotechnology Inc., Houston, TX, USA) at 4 °C overnight. Samples were then incubated with secondary antibodies at 1:200 dilution (polyclonal swine anti-rabbit, Dako Denmark) for 60 min and washed twice with rinse buffer before horseradish peroxidase-conjugated streptavidin (HRP-streptavidin) inoculation. All slices were dehydrated, mounted, scanned, and photographed with a Nikon ECLIPSE Ti-U microscope (Nikon Instruments Inc., Melville, NY, USA) under 100× magnification. Finally, five view images were quantified by Image J (version 1.50, National Institutes of Health, Bethesda, MD, USA) [57]. 

### 4.11. Statistical Analysis

All data are expressed as mean ± standard error. Comparisons between control and TET-treated groups were performed using one-way ANOVA. Tukey’s multiple comparisons test was performed. Statistical significance was assumed for *p*-values *<* 0.05.

## Figures and Tables

**Table 1:** The accurate *p*-value in Figure 3A is displayed. Here we summarized the *p*-value between control and two treatment groups of tumor volume.

Compares Group	TET 25 mg/kg	TET 50 mg/kg
Day 5		
Vs. Control	0.120	0.014
Vs. TET 25 mg/kg	--	0.640
Day 10		
Vs. Control	<0.0001	<0.0001
Vs. TET 25 mg/kg	--	0.030
Day 15		
Vs. Control	<0.0001	<0.0001
Vs. TET 25 mg/kg	--	<0.0001
Day 20		
Vs. Control	<0.0001	<0.0001
Vs. TET 25 mg/kg	--	<0.0001

Tukey’s multiple comparisons test was performed.

**Table 2:** The accurate *p*-value in Figure 3C is displayed. Here we summarized the *p*-value between control and two treatment groups of tumor weight.

Compares Group	TET 25 mg/kg	TET 50 mg/kg
Vs. Control	<0.0001	<0.0001
Vs. TET 25 mg/kg	--	<0.0001

Tukey’s multiple comparisons test was performed.

**Table 3:** The accurate *p*-value in Figure 4B is displayed. Here we summarized the *p*-value between control and two treatment groups of *Luc2* signal intensity.

Compares Group	TET 25 mg/kg	TET 50 mg/kg
Day 7		
Vs. Control	<0.0001	<0.0001
Vs. TET 25 mg/kg	--.	0.05
Day 14		
Vs. Control	<0.0001	<0.0001
Vs. TET 25 mg/kg	--	<0.0001
Day 21		
Vs. Control	<0.0001	<0.0001
Vs. TET 25 mg/kg	--	<0.0001

Tukey’s multiple comparisons test was performed.

## Data Availability

Datasets collected or analyzed during the current study are available from the corresponding author on request.
